# Junior to senior transition pathway in Italian Football: The rocky road to the top is not determined by youth national team’s selections

**DOI:** 10.1371/journal.pone.0288594

**Published:** 2023-07-18

**Authors:** Gennaro Boccia, Paolo Riccardo Brustio, Rocco Rinaldi, Ruggero Romagnoli, Marco Cardinale, Maria Francesca Piacentini

**Affiliations:** 1 Department of Clinical and Biological Sciences, University of Turin, Turin, Italy; 2 Neuromuscular Function Research Group, School of Exercise & Sport Sciences, University of Turin, Turin, Italy; 3 Human Movement and Health Sciences Università degli Studi di Roma Foro Italico, Rome, Italy; 4 Aspetar Orthopedic Hospital, Department of Research and Scientific Support, Doha, Qatar; 5 University College London, Institute of Sport Exercise and Health, London, United Kingdom; University of A Coruna: Universidade da Coruna, SPAIN

## Abstract

Football is a popular sport, but little is known about the youth-to-senior transition rates in elite players, particularly in large and successful countries. This study aims to investigate the youth-to-senior transition rate in the Italian national football team, both prospectively and retrospectively, and to explore if Relative Age Effects (RAEs) affect this transition. Data from 885 players selected in youth and senior Italian national teams between 2000 and 2021 were included in the study. For each player, the birthdate and the number of selection in Under 16, 17, 19, 21 and senior team was considered. The transition rate was determined by the number of youth players competing in the Senior National team (and vice versa), whilst birth quarter (Q) distributions with a chi-square goodness-of-fit test. Prospectively, the transition rate increased as age increased (i.e., from ~20% in U16 to ~50% in U19). Retrospectively, less than 10–20% of youth players were subsequently selected for the senior team. Data revealed a skewed birth date distribution in all age groups, and the RAEs magnitude decreased when age increased (i.e., ORs for Q1 vs Q4 was ~ 9 in U16 and ~ 1.7 in senior teams). Nevertheless, the RAE magnitude was smaller for successfully transitioned players. In conclusion, most players in the senior team were not previously selected for youth teams suggesting that junior international experience may not be a prerequisite for later success. Moreover, while the birthdate strongly influences the selection of youth national teams, its impact is less evident in the youth-to-senior transition.

## Introduction

In recent years, most sports governing bodies/federations have tried to develop effective strategies to identify youth athletes with the potential to become senior high-level athletes. The talent identification and selection process is complex, not straightforward, and highly challenging [1], especially considering that early success may not guarantee success at the senior level, as suggested in some sports [2–4]. In this regard, recent prospective and retrospective studies demonstrated that many successful young athletes do not necessarily appear among those who reach success during their senior career [2, 3, 5–9]. For example, only around 20% of elite international athletes who performed at the highest level during their youth could maintain that same level of excellence in their senior career [2, 4, 5, 7, 8].

Team sports have more complex parameters that define talent [1]. Successful athletes have advanced technical abilities, tactical awareness, and appropriate physicality for the sport performed. Additionally, the selection of players is also influenced by cognitive biases exhibited by social agents, such as coaches, who are involved in the selection process. This further amplifies the potential unreliability of selection decisions [10, 11]. Studies conducted on football players in different European countries showed uncertainty around the relevance of early recruitment relative to a successful transition [12–17]. Thanks to these studies, it seems evident that the experience gained from youth international teams is a limited predictor of senior success [16], the ability to obtain a professional contract [14] and may be linked to the age when players were selected [16]. A study on different European countries, including Scandinavian nations (i.e., Denmark, Norway, and Sweden), Belgium, Germany, and Portugal, highlighted that being included in Under 17 (U17) national selection negatively predicted subsequent participation in international football competitions. Also, the relationship between Under 19 (U19) selection and senior international participation was weak and increased with the selection at the Under 21 (U21) level [16]. Successful transition from youth-to-senior in football can also be measured by the ability of a player to obtain a professional contract in adulthood. Only 10% of U19 players in a German football academy successfully obtained professional contracts [18]. Studying the junior-elite football academy of the Scottish Football Association, Dugdale et al. [14] reported that only 10% of selected academy players reach senior success (i.e., obtain a professional contract). In Portuguese athletes, including football players, only a third of pre-junior athletes selected in youth national teams reappeared in the selections for adult athletes [15]. Overall, the professional academies showed a high turnover of youth players [18] mostly caused by repeated selection and de-selection procedures through childhood and youth rather than from early selection and long-term continuous nurture [12]. One possible explanation for this low successful youth-professional transition may be attributed to the fact that players’ identification was usually based more on players’ current performance than on their future potential [19]. Consequently, the emphasis of youth sport organisations towards short-term success inevitably influence athletes’ developmental pathways. This emphasis influences selection decisions and compromises future positive career outcomes both for selected and unselected (or de-selected) players.

The Relative Age Effects (RAEs) are among the most noticeable confounding factors affecting academies’ talent selection and development strategies [1], arising primarily from the interaction between the dates of selection and birthdates [3, 20, 21]. Additionally, RAEs are influenced by the choice of sports governing bodies/federations to group athletes according to (bi)annual age-group cohorts [20, 21]. Recent studies show that RAEs persist at professional and youth football levels independently of gender [3, 17, 22, 23]. Nevertheless, even if the prevalence of RAEs decreases as age increases [22, 23], relatively older players have more chances to be selected by elite teams, both at the young and senior levels [24]. For instance, depending on age group, 66–69% of the youth academy players in Germany, and even 72–81% of the German youth national players, are born in the year’s first half [25]. The bias in selecting individuals born earlier in the selection year may exist within male football academy structures but not at the amateur level [26]. The chronological difference among the same age groups may create performance disparities in favour of earlier-born athletes [27, 28]. Consequently, this gap created unequal opportunities and competition presence according to chronological age [29]. On the contrary, selection bias limits the possibility of potentially selecting talented athletes born late in the year and may result in premature de-selection and dropout [30]. Nevertheless, even if relatively older players are commonly overrepresented at the senior level [17, 22, 23], the transition from the academy to the professional level may be affected by a reversal effect where relatively younger players, once they enter the senior pathway, are more likely to achieve professional or international status [17, 31].

Considering the limited knowledge of the development of elite football players, we investigated the patterns of junior and senior national team selections in Italy. The first aim of the study was to calculate how many football players selected to represent Italy at young ages (i.e., youth national team) were also selected in senior national teams later in their careers. The secondary aim of this study was to verify whether RAEs affected the selection procedures and if it could explain the differences between successful and unsuccessful players.

## Materials and methods

This investigation focused on male football players of the youth and senior Italian national teams. All data are available in the public domain and were retrieved from the Italian Football Federation (FIGC) website (https://www.figc.it/it/nazionali/). We arbitrarily included in the analysis the players who were selected from 2000 to 2018 in the U16 (N = 426) and U19 (N = 570) national teams. We also included the players who played in the senior national teams from 2004 to September 30^th^ 2021 (N = 137). We restricted the analysis to the players born between 1983 and 1998 to consider only players with all their professional playing careers. The data collection consisted firstly in downloading the list of players selected for the matches played in the period considered (https://www.figc.it/it/nazionali/nazionali-in-cifre/elenco-gare/?squadraid=12). Afterwards, we searched the players in the FIGC database (https://www.figc.it/it/nazionali/nazionali-in-cifre/convocazioni-di-un-giocatore/?squadraId=12) and downloaded the number of selection from each official national team (i.e., U16, U17, U18, U19, U20, U21, and senior national teams). With this approach, we could reconstruct the complete list of selections in all junior and senior teams for each player. The birth date and playing position of each player were also recorded. Playing position was categorized into general positions (i.e., goalkeeper, defender, midfielder, and forward) based on the information provided by the FIGC database. After merging the databases and removing all duplicate names, the analysis was conducted on a sample of 885 players. All the study data were in the public domain. Therefore, informed consent was not required.

## Analysis

As previously reported [2, 3, 5–8], we adopted a prospective and retrospective approach to answering the first experimental question. For the prospective analysis, we calculated the transition rate as the frequency of players selected (at least once) for the youth national teams (i.e., U16 –U21) that were also selected (at least once) for the senior national team later in their career. For the retrospective approach, we calculated the transition rate as the frequency of players selected for the senior national team, which were also selected in youth national teams in the early stage of their careers. The transition rates were calculated prospectively and retrospectively using a binomial proportion confidence interval (95% CI). As the median of selections across all categories was 5, we also performed the same analysis using five annual selections as a cut-off for inclusion. Beyond the overall analysis, the players were sub-grouped for their positions (forward, midfielder, defender, goalkeeper).

To answer the second experimental question, the birth dates of each player were grouped in quartiles. The relatively older players were born in January–March = Quartile 1 (Q1); those between April and June = Quartile 2 (Q2); July–September = Quartile 3 (Q3); whilst the relatively youngest were those born between October and December = Quartile 4 (Q4). To identify RAEs, differences between observed and expected uniform quartile distributions (i.e., 25% for each quartile) [22] were assessed using Chi-Square Goodness of Fit tests (χ^2^), with P set at <0.05, and effect magnitudes determined by Cramer’s V. The threshold values for effect magnitudes were: 0.06 ≤ V for a trivial effect; 0.06 < V ≤ 0.17 for a small effect; 0.17 < V < 0.29 for a medium effect; and V ≥ 0.29 for a large effect. Odds ratios (ORs) and 95% confidence intervals [95% CIs] then identified discrepancies between Q1 vs Q4 and for Q1,2 vs Q3,4 (i.e., half-year distribution comparisons) [27]. The analyses were computed for the overall sample and separately for those who succeeded or failed to transition from junior to senior career. A successful transition indicates a player selected both in junior and senior categories. A failed transition indicates a player selected in the junior but not senior category. The Chi-Square test was furthermore computed for the subgroup of players (i.e., Only Senior subgroup) selected for senior but not for any youth team in U16, U17, and U18.

## Results

**Fig 1:**
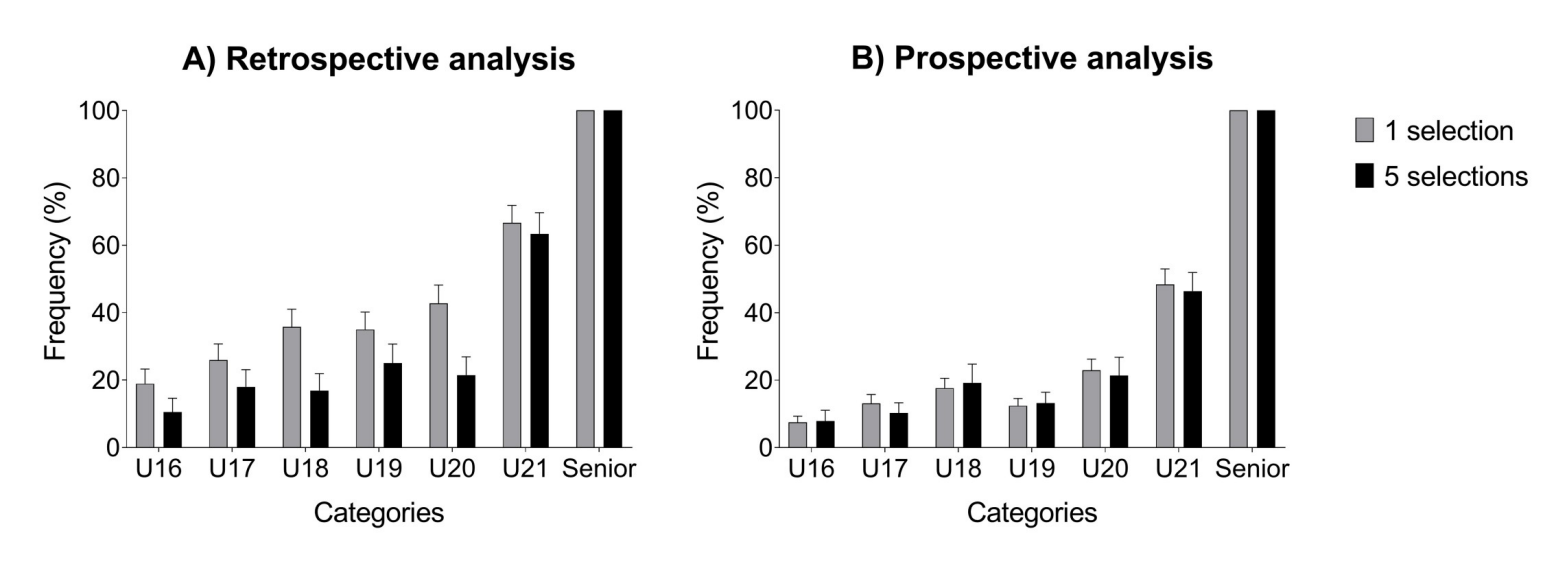
Junior to senior transition rates calculated in A) retrospective and B) prospective approach from U16 to U21 age group categories. Retrospective analysis reports how many players selected in the senior national team were already selected in junior categories. Prospective analysis reports how many players selected in the junior national teams were subsequently selected in the senior national team. The analysis was performed using two cut-off thresholds: one and five national team selection call-up for each category.

The retrospective analysis indicated that most players selected for the senior team had not been previously selected for junior teams (Fig 1A).

In fact, less than 20% of senior national team players were already selected in U16. Differently, 55% of senior national team players were already selected for the U19 team. The prospective analysis showed that most players selected in junior teams were not subsequently selected for the senior team (Fig 1B). Less than 10–20% of players selected in U16, U17, U18, and U19 national teams were subsequently selected for the senior national team.

The cut-off threshold for the number of selections slightly affected the transition rate in the retrospective analysis. When imposing a higher performance level (i.e., five selections vs one selection), the transition rate decreased by ~10–15% in all categories.

The playing position did not significantly affect the transition rate (see S1 Fig). However, on average, goalkeepers had the highest transition rate, while midfielders had the lowest.

**Fig 2:**
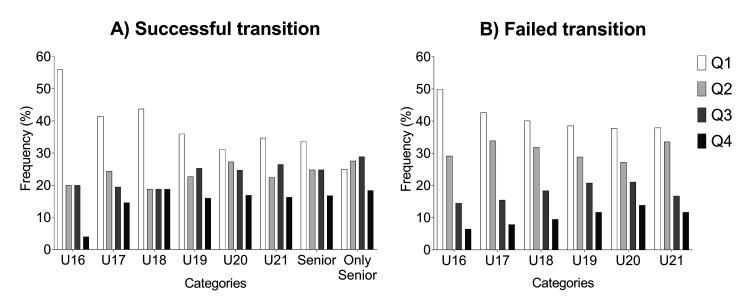
Birthdate quartile distributions for players who A) succeed and B) failed to transit from junior to senior national teams (considering one selection call-up). Overall, a pronounced asymmetry in quartile distribution can be seen favouring players born in the first part of the year (Q1 and Q2) compared to those born in the second part (Q3 and Q4). The RAEs decreased with age (from U16 to senior team). The players who successfully transitioned from junior to senior national teams (Panel A) were less affected by RAEs than those who failed to transit (Panel B). RAEs did not affect the players who reached the national teams only as senior national team players (after the U21 age limits), i.e., the Only Senior subgroup (Panel A).

S1 Table reports the age quartile distributions, including Chi-square statistics and ORs from U16 to Senior teams. Overall effect size decreased with age (i.e., from 0.4 in U16 to 0.2 in U21 and 0.1 in the Senior team). Quartile comparison ORs also decreased with age (i.e., Q1 vs Q4 was ~9 in U16, ~3.3 in U19, and ~1.7 in the senior team). The cut-off threshold for the number of selections did not affect RAEs: the effect sizes were similar in those selected at least once compared to those selected at least five times (S1 Table). The RAEs magnitude of those who successfully transitioned from junior to senior national teams (i.e., those selected both in junior and senior categories) was smaller than those who failed to transition (i.e., those selected in junior categories but not in the senior one) (See Fig 2). Lastly, RAEs did not affect the subgroup of players selected for senior national teams but that had never been selected for U16, U17, and U18 teams (P > 0.23, see S1 Table).

## Discussion

Since 2004, 1206 Italian male football players have been selected for youth and senior national teams. We calculated how many senior national players were selected in youth national teams (retrospective analysis) and how many junior national players were subsequently selected in senior national teams (prospective analysis). We also analysed the asymmetric birthdate distribution of national team players, representing the RAEs’ distribution. We found that: 1) less than 20% of players selected in senior national teams had already been selected in U16, while this prevalence increased up to 55% for U19 teams; 2) 9% and 15% of the players selected in U16 –U19 had subsequently been selected in the senior national teams, respectively; 3) the players born in the first quartile of the year were 8.3 times more represented than those born in the last one in U16 and 3.3 times in U19; 4) the subgroup of players that were selected for national teams solely in the adulthood was not affected by the RAEs.

From this analysis, we can conclude that in Italian Football, selection in a youth national team is not a prerequisite for success at achieving senior national team call-ups. In particular, when increasing the frequency of selections (i.e., one vs five selections at each age group), the proportion of players who succeeded in both junior and senior categories decreased by 15%. The analysis results in this cohort of players clearly suggest that even consistent representation at the national team level in youth might not translate into senior success in Italian Football. While the present study dealt with the Italian context, we can expect that the same trend would have been found, with minor differences, in other European countries. Many football players representing their national teams have never been selected before adulthood; therefore, it is likely that their talent (or performances) started to stand out only after complete maturation. There are exceptions to this rule if we consider that some of the most successful Football players of the modern era (e.g., Messi and Ronaldo) started representing their country at a young age. The findings about U16 challenge the convention that youth national teams increase exposure for young talents to mature then and produce success at the senior level. The analysis results on the U19 offer a partially different scenario: 55% of senior team players were already selected at the U19 level. Therefore, U19 teams’ selection may at least partially identify and nurture talent to achieve later success. Considering the findings of this cohort, national federations should reflect on their youth national teams’ pathways and maybe diversify the substantial investments to ensure that the players with the potential to represent the senior team are not overlooked at the youth level. This is easier said than done and not confined to Italy or Football. In fact, many studies reported the same trend in other countries and team sports [12, 14–16].

The prospective analysis showed that 9% and 15% (for U16 and U19, respectively) of players selected for the junior national teams joined the senior national team in adulthood. Optimistically, such a low transition rate could be seen as the result of a strategic approach adopted by the federation: many young players were “tested” during junior categories, whereas only a few of them were later confirmed for the senior category. However, this can only be partially supported by the number of players selected in U16 –U19 (426 and 570 players, respectively) and senior (~137 players), as analysed in this work. Despite more players joining the junior compared to the senior teams, 9–15% of the junior-to-senior transition rate may not represent a good return on investment. Overall, the present findings suggest that youth experience is a limited predictor of senior success [12, 14–16] and indicate a high turnover in national youth teams’ rosters [18]. The combination of the prospective and retrospective analysis showed that the selection strategies adopted at the youth level were linked to the determination of the players’ pool of the senior national teams only from the U19’s selection onwards. Evidently, the players joining the junior national teams were selected to win junior competitions instead of offering international experiences for the future generation of senior national players, which is expected with the demands for such teams to participate in international competitions. The prevalence of the RAEs in junior categories supports this observation.

RAEs confuse talent identification because relatively older players have more chances to be selected by national teams. For example, approximately 50% of U16 were born in Q1, whereas only 5% were in Q4 (Fig 2). It is possible to speculate that the chronological advance of players born in Q1 than in Q4 [32] creates a performance advantage, translating into more opportunities to be selected for junior national teams. Therefore, coaches and talent scouts may consciously or unconsciously select relatively older players because football players were traditionally selected based on a subjective analysis. For this reason, potentially talented players born late in the year have less chance of being selected in favour of potentially less talented players born early in the year purely because of their physicality [33]. This can explain the low youth-to-senior transition rate in the present study, similar to another recently published study [17]. To reinforce the hypothesis that players born earlier were erroneously considered destined for success, players who failed the junior-to-senior transition showed an even more pronounced RAE (Fig 2B). Even more interesting, players who joined the senior national team without any previous participation in youth national teams are unaffected by RAE. Data corroborates that RAEs magnitude further downsizes according to age and selection level [17, 22, 31]. When the effect of chronological age disappears from the selection policies, meaning that the selection process is undertaken at an older age, the birthdates of selected players are evenly distributed throughout the year. Overall, not controlling for the birthdate effect excludes late-born players from talent selection and decreases their chance to gain experience in an international context.

The present study has some limitations that should be underlined. We collected the junior data only relative to U16 and U19 teams. U16 represents the first age-group category selected for national teams. U19 represents the last age-group among the junior teams. Another relevant limitation is that the database does not differentiate between friendly and official competitions.

## Conclusion

In conclusion, our findings show a junior-to-senior transition rate in Italian football national teams of approximately 9–15%. This means that only a few players selected for junior national teams will join the senior national team later in their career. Therefore, the selection of the junior national team is an unreliable marker of future success in football. Furthermore, the chance of being selected for junior national teams is strongly affected by the birthdate: players born early in the calendar year have 3 to 8 times more chance to be selected than players born later in the year.

## Practical implication

- Football’s talent and development process should consider that less than 20% of players already selected in youth teams reach a high level during their senior career. This implies the necessity to explore alternative talent identification and development approaches. Practically, it is necessary to construct a more realistic expectation for future development players not only based on concurrent performance but on a long-term perspective.
- The present results indicate that the selection strategies for youth national teams are potentially misguided. This may create gaps in athletes’ developmental pathways, compromising future positive career outcomes for selected and unselected players. Again, this gap may be created by players’ chronological age, which appears to affect the selection process in the youth stages but is overlooked when the selection is completed during adulthood. Thus, policies should consider birthdate in the complexity of identification and development processes
- National Federations can use the present findings to review and benchmark their policies with realistic data on the long-term potential of their talents and their development system. In particular, Federations should apply strategies to include players born later in the year and/or late maturer athletes in expanded selections to provide more opportunities to compete internationally at an earlier age, possibly using ‘development squads’.

## Supporting information

S1 Fig Junior to senior transition rates calculated in retrospective and prospective approach from U16 to U21 age group categories according to play position. (TIF)

S1 Table Birthdate quartile distribution, chi-square value and odds ratio analysis. (DOCX)

S1 Data (TXT)
