# Supplementary figures and images for: Junior to senior transition pathway in Italian Football: The rocky road to the top is not determined by youth national team’s selections
Source: PLoS One. 2023 Jul 18;18(7):e0288594. doi: 10.1371/journal.pone.0288594 (PMC10353809; doi:10.1371/journal.pone.0288594)

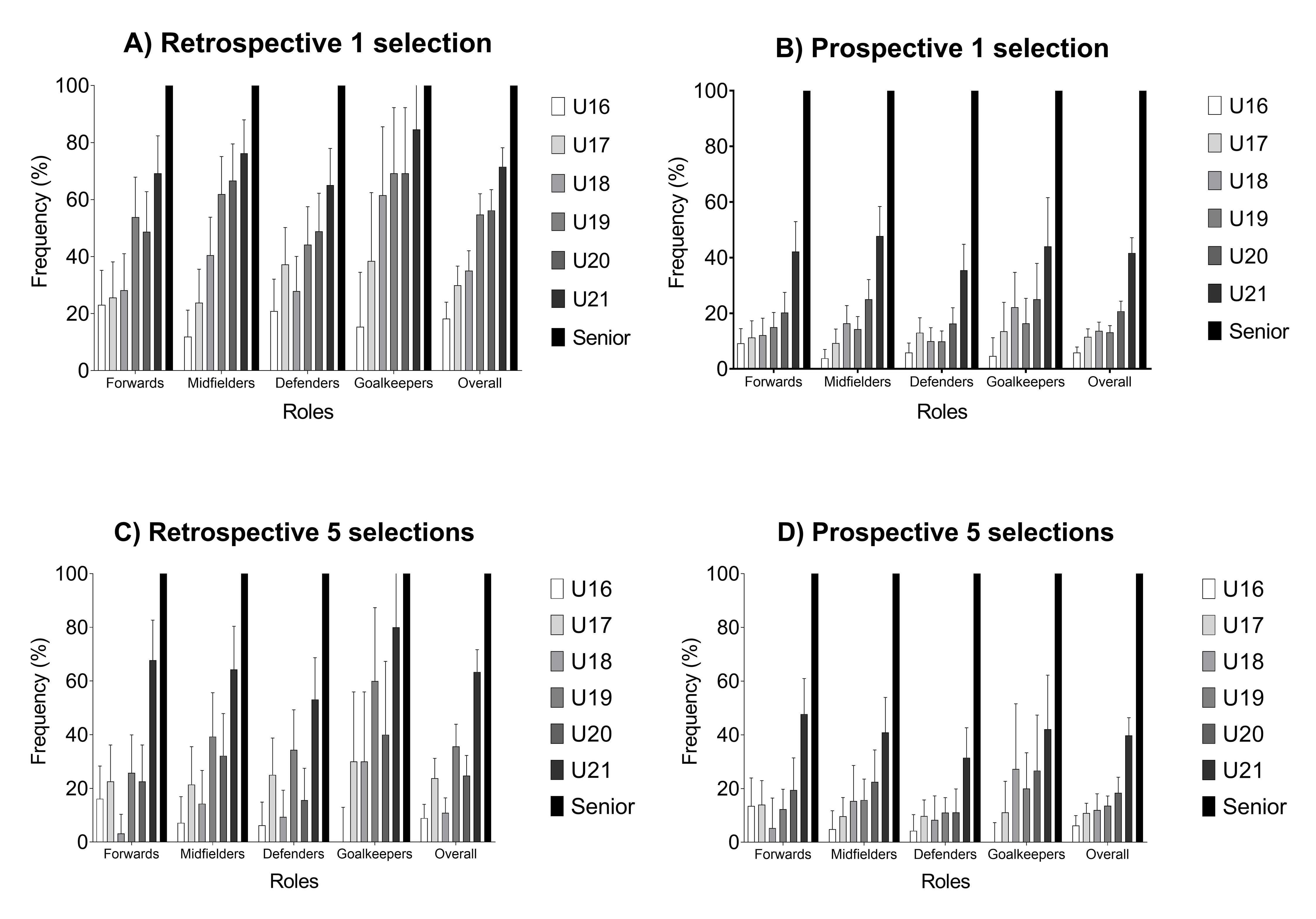

Supplement: S1 Fig — (TIF) [file pone.0288594.s001.tif]
