# Supplementary material for: Junior to senior transition pathway in Italian Football: The rocky road to the top is not determined by youth national team’s selections
Source: PLoS One. 2023 Jul 18;18(7):e0288594. doi: 10.1371/journal.pone.0288594 (PMC10353809; doi:10.1371/journal.pone.0288594)
Supplement: S1 Table — (DOCX) [file pone.0288594.s002.docx]

| Table 1. Birthdate quartile distribution, chi-square value and odds ratio analysis | | | | | | | | | | | | |
| --- | --- | --- | --- | --- | --- | --- | --- | --- | --- | --- | --- | --- |
| Age-group | Sub-groups | Total N | Q1 % | Q2 % | Q3 % | Q4 % | *χ^2^* | *P* | *V* | *ES category* | OR  Q1 VS Q4 | OR  Q1,2 VS Q3,4 |
| **One Convocation cut-off threshold** | | | | | | | | | | | | |
| U16 | Succeed | 25 | 56.0 | 20.0 | 20.0 | 4.0 | 15.6 | 0.001 | 0.45 | **Large** | 14.0 (2.0, 98.3) | 3.4 (1.2, 9.4) |
|  | Failed | 401 | 49.9 | 29.2 | 14.5 | 6.5 | 190.1 | <0.001 | 0.39 | **Large** | 8.0 (5.2, 12.1) | 3.9 (3.0, 5.1) |
|  | Overall | 426 | 50.2 | 28.6 | 14.8 | 6.3 | 204.3 | <0.001 | 0.40 | **Large** | 8.3 (5.5, 12.4) | 3.9 (3.0, 5.0) |
| U17 | Succeed | 41 | 41.5 | 24.4 | 19.5 | 14.6 | 7.6 | 0.010 | 0.25 | **Medium** | 2.8 (1.0, 8.2) | 2.0 (1.0, 2.1) |
|  | Failed | 316 | 42.7 | 33.9 | 15.5 | 7.9 | 106.3 | <0.001 | 0.33 | **Large** | 5.6 (3.5, 8.7) | 3.3 (2.5, 4.5) |
|  | Overall | 357 | 42.6 | 32.8 | 16.0 | 8.7 | 111.8 | <0.001 | 0.32 | **Large** | 5.0 (3.3, 7.6) | 3.1 (2.4, 4.1) |
| U18 | Succeed | 48 | 43.8 | 18.8 | 18.8 | 18.8 | 10.2 | 0.016 | 0.27 | **Medium** | 2.3 (0.9, 5.9) | 1.6 (0.8, 3.2) |
|  | Failed | 304 | 40.1 | 31.9 | 18.4 | 9.5 | 74.5 | <0.001 | 0.28 | **Medium** | 4.3 (2.8, 6.7) | 2.6 (2.0, 3.5) |
|  | Overall | 352 | 40.6 | 30.1 | 18.5 | 10.8 | 80.3 | <0.001 | 0.28 | **Medium** | 3.9 (2.6, 5.8) | 2.5 (1.9, 3.2) |
| U19 | Succeed | 75 | 36.0 | 22.7 | 25.3 | 16.0 | 7.1 | 0.068 | 0.18 | Medium | 2.3 (1.0, 5.2) | 1.4 (0.8, 2.5) |
|  | Failed | 495 | 38.6 | 28.9 | 20.8 | 11.7 | 86.9 | <0.001 | 0.24 | **Medium** | 3.4 (2.4, 4.7) | 2.1 (1.7, 2.6) |
|  | Overall | 570 | 38.2 | 28.1 | 21.4 | 12.3 | 91.8 | <0.001 | 0.23 | **Medium** | 3.2 (2.4, 4.3) | 2.0 (1.6, 2.5) |
| U20 | Succeed | 77 | 31.2 | 27.3 | 24.7 | 16.9 | 3.9 | 0.273 | 0.13 | Small | 1.9 (0.9, 4.2) | 1.4 (0.8, 2.4) |
|  | Failed | 294 | 37.8 | 27.2 | 21.1 | 13.9 | 40.8 | <0.001 | 0.21 | **Medium** | 2.8 (1.8, 4.2) | 1.9 (1.4, 2.5) |
|  | Overall | 371 | 36.4 | 27.2 | 21.8 | 14.6 | 43.1 | <0.001 | 0.20 | **Medium** | 2.6 (1.8, 3.7) | 1.8 (1.4, 2.3) |
| U21 | Succeed | 98 | 34.7 | 22.4 | 26.5 | 16.3 | 7.9 | 0.048 | 0.16 | **Small** | 2.2 (1.1, 4.3) | 1.4 (0.9, 2.2) |
|  | Failed | 137 | 38.0 | 33.6 | 16.8 | 11.7 | 29.3 | <0.001 | 0.27 | **Medium** | 3.3 (1.8, 6.2) | 2.6 (1.7, 4.0) |
|  | Overall | 235 | 36.6 | 28.9 | 20.9 | 13.6 | 31.5 | <0.001 | 0.21 | **Medium** | 2.8 (1.8, 4.4) | 1.9 (1.4, 2.7) |
| Senior | Overall | 137 | 33.6 | 24.8 | 24.8 | 16.8 | 9.0 | 0.029 | 0.15 | **Small** | 2.0 (1.1, 3.6) | 1.4 (1.0, 2.1) |
|  | Only Senior | 76 | 25.0 | 27.6 | 28.9 | 18.4 | 1.8 | 0.610 | 0.09 | Small | 1.5 (0.7, 3.5) | 1.1 (0.6, 2.0) |
| *Notes*: Q1, first quartile percentage; Q2, second quartile percentage; Q3, third quartile percentage; Q4, fourth quartile percentage; χ^2^, Chi-square value; V, Cramer’s V effect size. When the χ^2^ was statistically significant (P<0.05) the effect size category is highlighted in bold. OR, odds ratio and 95% conﬁdence intervals (95% CI); Q1-Q4, ﬁrst versus last quartile; Q1,2-Q3,4 ﬁrst versus last the half year’s distribution. Sub-groups refer to players who succeed or fail the transition from junior convocation to senior. See the methods section for details. | | | | | | | | | | | | |
